# Stressful life events and current psychological distress are associated with self-reported hypertension but not with true hypertension: results from a cross-sectional population-based study

**DOI:** 10.1186/1471-2458-8-357

**Published:** 2008-10-15

**Authors:** Felipe Sparrenberger, Sandra C Fuchs, Leila B Moreira, Flávio D Fuchs

**Affiliations:** 1 Department of Medicine, Universidade Regional de Blumenau, Blumenau, Brazil; 2 Department of Social Medicine, Universidade Federal do Rio Grande do Sul, Porto Alegre, Brazil; 3 Division of Clinical Pharmacology, Hospital de Clínicas de Porto Alegre, Universidade Federal do Rio Grande do Sul, Porto Alegre, Brazil; 4 Division of Cardiology, Hospital de Clínicas de Porto Alegre, Universidade Federal do Rio Grande do Sul, Porto Alegre, Brazil

## Abstract

**Background:**

The evidence linking stress to hypertension has been scarcely documented in population-based studies.

**Methods:**

Participants were selected through a multi-stage probability sampling and interviewed at home, being submitted to measures of demographics, anthropometrics, blood pressure (BP), and risk factors for hypertension. Hypertension was defined as BP ≥ 140/90 mm Hg or use of BP-lowering drugs or as self-reported hypertension. Stressful life events were investigated through an inventory of nine major life events occurring in the year preceding the interview. Psychological distress was evaluated through a facial scale of expression of emotion in the last month.

**Results:**

In the total, 1,484 adult individuals were investigated. Prevalence of hypertension was lower in individuals who reported any stressful life event in comparison with individuals who did not reported an event (34.3 versus 44.2%, P < 0.01), such as relative or friend death, loss of job, divorce, violence and migration. There was a trend for higher prevalence of hypertension in individuals with higher psychological distress in the last month, which was not longer significant after adjustment for confounding. In contrast, individuals who self-reported hypertension, but actually had normal blood pressure and were not using antihypertensive medication, reported higher numbers of stressful events.

**Conclusion:**

Recent stressful life events and current psychological distress are not associated with hypertension. Associations between stress events and distress with self-reported hypertension are not intermediated by effects of stress on blood pressure, and may be ascribed to negative feeling about disease and not to the disease itself.

## Background

Stressful life events have been long associated with hypertension [1-3], but the independence and strength of this association have been disputed [3-5]. Most evidence came from experiments in animal models [6], clinical experiments [7] and from the assessment of the association between exposure to stressors, such as job stress [8], catastrophes [9], and blood pressure measurement [10], with blood pressure. The findings from epidemiological surveys have not been homogeneous regarding the association between the occurrence of acute stressful events and sustained elevation of blood pressure [11,12]. Differences in study design, sampling criteria, population surveyed, definition of stress events, and hypertension, may account for the discrepancy of findings [12,13]. In some studies, the investigation of the association between stress and hypertension was a secondary objective, and stress or blood pressure were not directly determined.

In this prospective planned, population-based survey, we tried to circumvent some limitations of epidemiological studies, investigating if current psychological distress and stressful life events are associated with higher blood pressure and hypertension in individuals living in communities.

## Methods

### Design, participants and data collection

This study is a report of a large cross-sectional, population-based study aiming to investigate several hypotheses related to cardiovascular disease in Porto Alegre, the Study of Obesity and Risk Factors (SOFT study). Participants were identified through cross-sectional, population-based, multi-stage probability sampling and interviewed at home after giving informed consent. The study was approved by the Ethics Committee of Hospital de Clínicas of Porto Alegre, which is accredited by the Office of Human Research Protections as an Institutional Review Board. A sampling of individuals aged 18 to 60 years old (none to two individuals in each household according to the number of individuals living at each domicile) plus all residents older than 60 years were interviewed, in order to have an overrepresentation of elderly individuals. Data were collected with a structured and pre-tested questionnaire, which included assessment of demographic characteristics, years at school, familial history of hypertension, smoking, drug treatments, and various questions pertaining to several objectives of the SOFT study. Physical activity was assessed through the International Physical Activities Questionnaire [14]. The type, quantity, and frequency of alcoholic beverage consumption were assessed and the average daily alcohol intake was calculated. Anthropometric measurements were carried out with participants wearing light clothing and no shoes. The field work was done between 2005 and 2007.

### Blood pressure measurement and definition of outcome variables

Sitting blood pressure was determined four times during the interview with an automatic and validated device, Omron 705 CP [15]. The average of these measurements was employed in analysis. Hypertension was defined by blood pressure ≥ 140/90 mmHg or use of blood pressure-lowering medication. In addition, we explored the association of stress events with the following outcomes: self-reported hypertension, when the participant reported having the diagnosis of hypertension done by a physician or a nurse, independently of the blood pressure values measured in the interview or use of blood pressure drugs; true awareness, when the awareness combined with the diagnosis of hypertension was confirmed by blood pressure or use of blood pressure drugs; false awareness, when self-reported hypertension was not confirmed by blood pressure or use of blood pressure drugs; and high blood pressure, when the mean of four measurements was = 140/90 mmHg, independently of awareness of hypertension or use of blood pressure drugs.

### Measurements of stressful life events and other exposures

The questionnaire investigated the occurrence of nine stressor exposures events derived from seven questions in the year preceding the interview (Table 1). A scale of faces [16] was employed to evaluate the predominant mood in the in the last month, as a surrogate of the intensity of psychological distress. Each face consists of a circle with eyes that do not change and a mouth that varies from a smile of almost a half-circle to a similar half-circle upside down, representing variation of mood from extremely content to extremely discontent. Participants were asked about which face better represented his/her feelings for the most part of the last 30 days.

**Table 1:** Stressful life events in the last year

1) Did someone in your family or beloved to you die? How many? What was the kinship of this relative/beloved person?
2) Do you have anyone with a serious illness living at your home?
3) Were you fired?
4) Have you divorced or finished as stable relationship?
5) Did you have any serious accident that needed care in an emergency room or in hospitalization?
6) Did you suffer a physical violence?
7) Did you if move to Porto Alegre? What is your feeling about this move?

### Sample size calculation

The sample size of the SOFT study, of approximately 3,000 individuals, was calculated to grant enough power to test several prospectively planned hypotheses of the study. The present analysis was done with the first 1,474 enrolled adults. This sample size assumed a prevalence of hypertension of 30% among non-exposed and a relative risk of 1.25 (25% increase of risk among exposed), for a P alpha of 0.05 and a power of 85%.

### Statistical analysis

The characteristics of the sample were described by means ± SD or frequency and proportions. Blood pressure of individuals with and without stressor exposures and categories and in the categories of the scale of faces was compared by means of an analysis of covariance (ANCOVA), adjusting for age. Prevalence of hypertension, awareness of hypertension and high blood pressure by the stressor exposures was tested by Chi-square. A logistic regression model was used to control for confounding of the association between stressful life events and psychological distress evaluated by the scale of faces and hypertension, separately by gender. Risk factors for hypertension were included in the model. Analyses were done in the SPSS, version 14.0.

## Results

Table 2 presents demographic and anthropometric characteristics of the study sample, together with risk factors for hypertension, blood pressure and the proportion of individuals with hypertension. Most participants were women and the mean body mass index was within the overweight range. Measures of psychological distress in the whole sample are presented in table 3. About a third of the individuals had at least one stressful life event in the last year, mostly a death of a next of kin or a friend, severe illness in the family and loss of job. Some individuals had more than one event. The rate of total events by individual was 0.5 ± 0.8. The corresponding rate for deaths was 0.2 ± 0.4 per individual. Despite the high frequency of a major life event in the preceding year, 73.8% of the individuals were content, very content or extremely content in the month preceding the interview.

**Table 2:** Selected characteristics of the study sample (N = 1484)

Characteristics	
Age, years	48.8 ± 19.3
Women	869 (58.6%)
White skin color	1090 (73.5%)
Years at school	8.9 ± 4.7
Strong parental history of hypertension (both parents)	164 (11.1%)
Body mass index, Kg/m^2^	26.6 ± 5.4
Alcohol abuse*	145 (9.8%)
Physical activity (IPAQ)	
Low	494 (33.3%)
Moderate	527 (35.5%)
High	463 (31.2%)
Systolic blood pressure, mmHg	126.8 ± 22.0
Diastolic blood pressure, mmHg	76.9 ± 12.1
Hypertension**	600 (40.4%)
Aware of hypertension	489 (33%)
Blood pressure ≥ 140/90 mmHg	394 (26.5%)

Values are mean ± SD or number (percentage).

* alcohol abuse ≥ 30 g/day (men)/15 g/day (women)

** Blood pressure ≥ 140/90 mmHg or use of blood pressure-lowering medication

**Table 3:** Exposure to stressful life events and to psychological *distress *evaluated by the faces scale in the study sample (N = 1484)

Characteristics	N (%)
Any event*	571 (38.5)
Any death**	214 (14.4)
Major illness in the family	149 (10.0)
Loss of job	145 (9.8)
Death in the family	143 (9.6)
Divorce	90 (6.1)
Severe accident	71 (4.8)
Close friend death	68 (4.6)
Violence	41 (2.8)
Migration	34 (2.3)
Death of spouse	14 (0.9)
Number of deaths	
No death	1278 (85.6)
1 death	179 (12.0)
2 death	35 (2.3)
Number of events	
No events	913 (61.5)
1 event	407 (27.4)
2 events	127 (8.6)
3 or more events	37 (2.5)
Faces	
Extremely content	259 (17.5)
Very content	458 (30.9)
Content	377 (25.4)
Neutral	159 (10.7)
Discontent	114 (7.7)
Very discontent	57 (3.8)
Extremely discontent	60 (4)

Values are mean ± SD or number (percentage).

* One or more events.

** One or more deaths

Blood pressure was lower in individuals who had some stressful life event in the last year. For example, systolic and diastolic blood pressure of individuals that reported any life event were lower than blood pressure of those that did no report an event (124.2 ± 21.0 mmHg and 76.3 ± 12.0 mmHg versus 128.4 ± 22.4 mm Hg and 77.2 ± 12.2 mmHg, respectively, P < 0.01). These differences were no longer significant after adjustment for age (Table 4). No consistent association between the categories of the faces scale and blood pressure was observed (Table 4).

**Table 4:** Means of systolic and diastolic blood pressure* (mmHg) by stressor exposures and psychological distress evaluated by the faces scale (N = 1477)

Characteristic		N	Systolic BP	P	Diastolic BP	P
Any event	No	907	127.4 ± 0.7	0.12	76.9 ± 0.4	0.87
	Yes	570	125.8 ± 0.8		76.8 ± 0.5	
Any death	No	1264	127.0 ± 0.6	0.43	76.8 ± 0.3	0.62
	Yes	213	125.8 ± 1.4		77,3 ± 0.8	
Major illness in the family	No	1328	127.0 ± 0.6	0.31	76.9 ± 0.3	0.71
	Yes	149	125.2 ± 1.6		76.5 ± 1.0	
Loss of job	No	1332	127.0 ± 0.6	0.37	77.0 ± 0.3	0.38
	Yes	145	125.3 ± 1.7		76.0 ± 1.0	
Death in the family	No	1477	126.9 ± 0.6	0.53	76.8 ± 0.3	0.50
	Yes	143	125.8 ± 1.7		77.5 ± 1.0	
Divorce	No	1387	127.0 ± 0.5	0.19	77.0 ± 0.3	0.25
	Yes	90	124.0 ± 2.2		75.5 ± 1.3	
Severe accident	No	1406	127.0 ± 0.5	0.19	76.9 ± 0.3	0.80
	Yes	71	123.7 ± 2.4		76.5 ± 1.4	
Close friend death	No	1410	126.8 ± 0.5	0.78	76.8 ± 0.3	0.96
	Yes	67	126.1 ± 2.5		77.0 ± 1.4	
Violence	No	1436	126.8 ± 0.5	0.84	76.9 ± 0.3	0.98
	Yes	41	126.2 ± 3.2		76.9 ± 1.8	
Migration	No	1443	126.8 ± 0.5	0.92	76.9 ± 0.3	0.74
	Yes	34	117.2 ± 3.5		77.6 ± 2.0	
Death of spouse	No	1334	126.9 ± 0.5	0.10	76.9 ± 0.3	0.05
	Yes	14	117.9 ± 5.4		70.6 ± 3.1	
Faces				0.06		0.29
Extremely content		259	130.4 ± 1.2		78.2 ± 0.7	
Very content		455	126.8 ± 0.9		76.4 ± 0.6	
Content		377	125.3 ± 1.0		77.1 ± 0.6	
Neutral		158	125.7 ± 1.6		75.8 ± 0.9	
Discontent		113	126.6 ± 1.9		76.0 ± 1.1	
Very discontent		55	124.0 ± 2.7		75.9 ± 1.6	
Extremely discontent		60	126.6 ± 2.6		78.3 ± 1.5	

Values are mean ± standard error

* adjusted for age

Table 5 shows the association between stressful events in the last year and current distress with hypertension, self-reported hypertension and high blood pressure. Individuals who reported any death among relatives and friend in the last year had higher prevalence of hypertension defined by any criterion. The opposite occurred with loss of job, divorce, and migration. The categories of the faces scale were not consistently associated with hypertension and the proportion of individuals with high blood pressure, but presented a linear trend for an association with self-reported hypertension.

**Table 5:** Prevalence of true hypertension (BP ≥ 140/90 mmHg or use of BP drugs), awareness of hypertension and BP ≥ 140/90 mmHg by the occurrence of stressor exposures and psychological distress evaluated by the faces scale (N = 1484)

Characteristics		N	True hypertension* N = 600 (40.4%)	P	Awareness of hypertension N = 489 (33%)	P	BP ≥ 140/90 mmHg N = 394 (26.5%)	P
Any event	No	913	404 (44.2)	< 0.01	317 (34.7)	0.07	267 (29.2)	< 0.01
	Yes	571	196 (34.3)		172 (30.1)		127 (22.2)	
Any death	No	1270	495 (39.0)	< 0.01	404 (31.8)	0.02	328 (25.8)	0.12
	Yes	214	105 (49.1)		85 (39.7)		66 (30.8)	
Major illness	No	1335	548 (41.0)	0.15	438 (32.8)	0.73	362 (27.1)	0.14
	Yes	32	52 (34.9)		51 (34.2)		32 (21.5)	
Loss of job	No	1339	569 (42.5)	< 0.01	459 (34.3)	< 0.01	373 (27.9)	< 0.01
	Yes	145	31 (21.4)		30 (20.7)		21 (14.5)	
Death in the family	No	1341	531 (39.6)	0.05	433 (32.3)	0.10	349 (26)	0.16
	Yes	143	69 (48.3)		56 (39.2)		45 (31.5)	
Divorce	No	1394	583 (41.8)	< 0.01	473 (33.9)	< 0.01	382 (27.4)	< 0.01
	Yes	90	17 (18.9)		16 (17.8)		12 (13.3)	
Severe accident	No	1413	576 (40.8)	0.24	472 (33.4)	0.10	378 (26.8)	0.43
	Yes	71	24 (33.8)		17 (23.9)		16 (22.5)	
Friend death	No	1416	563 (39.8)	< 0.01	459 (32.4)	0.05	372 (26.3)	0.27
	Yes	68	37 (54.4)		30 (44.1)		22 (32.4)	
Violence	No	1443	592 (41.0)	< 0.01	480 (33.3)	0.13	389 (27)	0.04
	Yes	41	8 (19.5)		9 (22.0)		5 (12.2)	
Migration	No	1450	594 (41.0)	< 0.01	482 (33.2)	0.12	390 (26.9)	0.05
	Yes	34	6 (17.6)		7 (20.6)		4 (11.8)	
Death of spouse	No	1470	595 (40.5)	0.72	485 (33.1)	0.73	390 (26.5)	0.86
	Yes	14	5 (35.7)		4 (28.6)		4 (26.5)	
Faces				0.03**		< 0.01**		0.92**
Extremely content		259	112 (43.2)		85 (32.8)		79 (30.5)	
Very content		458	160 (34.9)		123 (26.9)		108 (23.6)	
Content		377	156 (41.4)		123 (32.6)		104 (27.6)	
Neutral		159	62 (39.0)		54 (34.05)		41 (25.8)	
Discontent		114	52 (45.6)		45 (39.5)		28 (24.6)	
Very discontent		57	24 (42.1)		25 (43.9)		17 (29.8)	
Extremely discontent		60	34 (56.7)		34 (56.7)		17 (28.3)	

* Includes individuals aware of diagnosis with uncontrolled BP and individuals unaware of the diagnosis

** P for linear trend

Table 6 presents the odds ratio for hypertension stratified by gender and for the whole sample, adjusting for age, skin color, physical activity, alcohol abuse, body mass index, strong parental history of hypertension and education. The trends observed in the bivariate analysis (table 5) disappeared, either for stress events in the last year as well for the categories of faces scale.

**Table 6:** The association between stressor exposures and psychological distress evaluated by faces scale with hypertension (OR and 95% CI)

Exposure	Men *	P	Women *	P	Overall**	P
Any event	0.7 (0.5–1.1)	0.31	0.8 (0.6–1.2)	0.15	0.8 (0.6–1.0)	0,10
Any death	1.2 (0.7–2.1)	0.51	1.1 (0.7–1.8)	0.60	1.2 (0.8–1.7)	0.33
Major illness in the family	0.6 (0.3–1.1)	0.11	0.9 (0.5–1.6)	0.78	0.8 (0.5–1.2)	0.23
Loss of job	0.7 (0.5–1.2)	0.69	0.7 (0.3–1.4)	0.35	0.7 (0.5–1.2)	0.24
Familiar death	0.8 (0.4–1.6)	0.59	1.4 (0.8–2.6)	0.18	1.2 (0.8–1.8)	0.44
Divorce	2.0 (0.8–5.1)	0.14	0.5 (0.2–1.3)	0.17	0.9 (0.5–1.7)	0.81
Severe accident	0.7 (0.3–1.7)	0.40	0.9 (0.4–2.1)	0.82	0.8 (0.4–1.5)	0.47
Close friend death	2.0 (0.8–5.6)	0.16	1.1 (0.5–2.4)	0.19	1.5 (0.8–2.7)	0.25
Violence	0.3 (0.1–1.1)	0.06	0.7 (0.2–3.0)	0.67	0.4 (0.2–1.1)	0.07
Migration	0.4 (0.1–1.7)	0.24	1.1 (0.3–4.9)	0.88	0.7 (0.3–1.9)	0.47
Death of spouse	2.3 (0.2–26.2)	0.52	0.3 (0.1–1.1)	0.07	0.4 (0.1–1.6)	0.21
Number of deaths		0.41		0.87		0.58
No death	1		1		1	
1 death	1.1 (0.6–2.1)		1.3 (0.8–2.2)		1.3 (0.9–1.9)	
2 death	2.1 (0.4–11.2)		0.7 (0.3–1.9)		1.4 (0.4–2.5)	
						
Number of events		0.24		0.37		0.16
No event	1		1		1	
1 event	0.7 (0.5–1.1)		0.9 (0.6–1.3)		0.8 (0.6–1.1)	
2 events	0.8 (0.4–1.7)		0.7 (0.4–1.3)		0.8 (0.5–1.2)	
More than 3 events	0.7 (0.1–3.9)		1.2 (0.3–4.4)		1.0 (0.3–2.6)	
Faces		0.18		0.40		0.18
Extremely content	1		1		1	
Very content	0.6 (0.4–1.1)		0.8 (0.5–1.4)		0.7 (0.5–1.0)	
Content	1.3 (0.7–2.3)		0.9 (0.5–1.6)		1.1 (0.7–1.6)	
Neutral	0.8 (0.4–1.7)		0.7 (0.3–1.4)		0.9 (0.5–1.3)	
Discontent	1.3 (0.5–3.4)		1.0 (0.5–2.0)		1.1 (0.6–1.9)	
Very discontent	0.5 (0.1–1.9)		0.8 (0.3–1.9)		0.7 (0.3–1.4)	
Extremely discontent	2.4 (0.6–8.9)		1.8 (0.7–4.1)		1.8 (0.9–3.6)	

*adjusted for age, skin color, physical activity, alcohol abuse, body mass index, strong parental history and education

** adjusted for gender, age, skin color, physical activity, alcohol abuse, body mass index, strong parental history and education.

**Figure 1:**
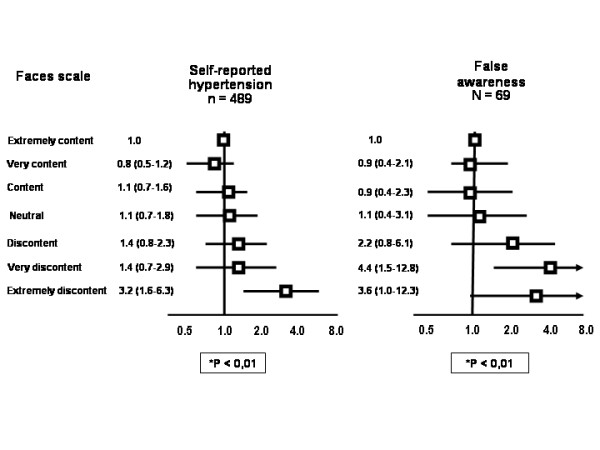
**The association between self-report hypertension and false awareness of hypertension with psychological distress evaluated by faces scale (OR and 95% CI, adjusted for gender, age, skin color, physical activity, alcohol abuse, body mass index, strong parental history and education).** *P for trend

**Figure 2:**
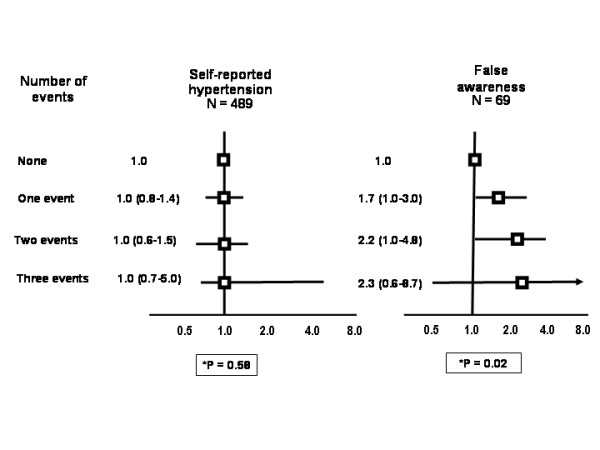
**The association between self-reported hypertension and false awareness of hypertension (reported hypertension not confirmed by BP or use of BP drugs) with the number of stressor exposures in the last year (OR and 95% CI, adjusted for gender, age, skin color, physical activity, alcohol abuse, body mass index, strong parental history and education).** *P for trend

Current psychological distress (figure 1) remained associated with self-reported hypertension in the multivariate analysis, particularly when the diagnosis was not confirmed by blood pressure measurement or use of blood pressure-lowering drug (false awareness). The number of stressful life events in the last year was associated only with the false awareness of hypertension (figure 2). Risk ratios for having blood pressure ≥ 140/90 mmHg, irrespective of awareness or use of blood pressure-lowering drugs, were all below 1.0 but not significant for the several categories of faces scale and the number of life events in the last year. These analyses were run separately by gender, and the estimates did not change substantially.

## Discussion

In this population-based cross-sectional study we were able to describe the prevalence of hypertension, the frequency of individuals with stressful life events in the last year and the current psychological distress evaluated by faces scale in a large sample of individuals living in communities. The estimates are not representative of the whole city in view of the oversampling of elderly individuals, but provided enough power to test the association between measures of stress and measures of hypertension. Overall, there was no association between any measure of stress and hypertension diagnosed by high blood pressure or use of blood pressure-lowering drugs. Psychological distress evaluated by faces scale, however, was associated with self-reported hypertension. Individuals that had a higher number of stressful life events in the last year reported hypertension not confirmed (false awareness) more frequently.

The belief that psychological factors affect long-term blood pressure regulation dates back to the early 20th century, since the classic hypothesis of Alexander linking repressed hostility to hypertension [17]. The belief that stress is a cause of high blood pressure and cardiovascular disease at all is deeply-rooted among populations. In a population survey in Canada, 44% of the participants believed that stress/worry was the major cause of cardiovascular disease [18]. Studies of the association between stress and hypertension have showed variable results. Most studies have identified positive trends [19,20], but even inverse associations have been reported [12,21]. The novel finding of our investigation was the contrasting association between self-reported hypertension and true hypertension with stress events and psychological distress evaluated by the faces scale.

The observation that young individuals with high blood pressure reported fewer life events was already reported by Theorell et al in a Sweden [22]. The lack of sensitivity to the environment seems to be a consistent trait in people who develop hypertension. In a laboratory study, subjects who had a positive family history of hypertension and who exhibited a personality pattern that included denial and unwillingness to admit to neurotic feelings or aggressiveness exhibited higher BP responsiveness during stress periods than subjects who had a negative family history [23]. A low sensitivity to pain has been also demonstrated in patients with hypertension [24]. We identified that individuals with high blood pressure had lower prevalence of migraine in a population-based study [25]. In the cohort of Nord-Trondelag there was a strong linear trend (P < .001) of decreasing prevalence of chronic musculoskeletal complaints with increasing BP values [26]. The notion that individuals with hypertension have some insensitivity to environmental stimulus is strengthened by studies that compared objective versus subjective reporting of stressful conditions. Objective stressors, such as job barriers and time pressure were significantly associated with hypertension, but not self-reported stressors at the individual level [27].

Additionally, the association between stress and hypertension reported in some studies may in fact be ascribed to the negative feeling about disease and not to the disease itself. Awareness of hypertension status (irrespective of actual blood pressure) appears to be associated with increased perceptions of psychological and physical symptoms [21]. In a meta-analysis, Jorgensen et al [28] concluded that hypertension is inversely associated with negative emotions in individuals who were unaware of the diagnosis and directly associated when they were aware of their blood pressure level. Other explanations for these associations have been proposed, such as that these individuals would look for health care more frequently, having a higher probability of having a diagnosis of hypertension. Our findings are in accordance with these interpretations, suggesting that the results of cohort studies that had used self-reported hypertension may be flawed [29,30].

Our study has some limitations that deserve mention. First, the cross-sectional design does not preclude an inverse causality, despite the fact that the exposures to stress events were evaluated retrospectively. This aspect would influence the association of stress with self-reported hypertension, but not with true hypertension, which was not associated with stress events and categories of faces scale. Second, scale of faces has not been commonly used in the evaluation of the association of distress events and hypertension. The association of more negative categories of the faces scale with stress life events, however, was reported before [31]. Third, we used a relatively small number of major stress events, since a middle-range of from 30 to 50 items seems both optimal in terms of predictive power and efficiency in terms of use of time and space [32]. The events that we investigated, however, were those more important and expectedly associated with profound consequences on cardiovascular regulation. Fourth, the exposure of individuals to stress events could result in hypertension in the future, an association that would be captured only in a longitudinal study. It is unlikely, however, that the absence of an association between stress events and current distress with blood pressure and hypertension would result in hypertension thereafter. Fifth, we did not adjust for the overrepresentation of elderly participants, therefore our estimates of prevalence of hypertension and stress does not apply to the whole city. The oversampling of elderly individuals, however, did not influence the direction and strength of the associations, since they were independent of age in the multivariate analysis. Sixth, the measurement of blood pressure in only one visit did not capture the usual blood pressure of the participants. This is, however, a conservative bias, since the alert reaction of some individuals to blood pressure determination would artificially increase blood pressure. Moreover, blood pressure was measured four times, with an automatic validated device, at home and not at office. Since blood pressure was measured at home it could not represent the values obtained in other environments, such as medical offices and at job. Therefore, our classification of false awareness of hypertension may reflect the discrepancy between office and home measurements. And finally, we did not study the risk of more chronic stress events [33] and daily and within-day events [34].

## Conclusion

Selected stressful life events in the last year and current psychological distress evaluated by the faces scale are not associated with hypertension in individuals living in communities. The association between stress events in the last year and current psychological distress with self-reported hypertension, together with the absence of association with high blood pressure, suggests that the reported associations between stress and reported hypertension are not intermediated by effects of stress on blood pressure.

## Competing interests

The authors declare that they have nocompeting interests.

## Authors' contributions

SCF and LBM conceived, designed and coordinated the study, and prepared the questionnaires. FS directed part of the field work. FS and FDF performed the statistical analysis and prepared the drafts of the manuscript. All authors read and approved the final manuscript.

## Pre-publication history

The pre-publication history for this paper can be accessed here:


